# The Role of Polyphenols in Abiotic Stress Tolerance and Their Antioxidant Properties to Scavenge Reactive Oxygen Species and Free Radicals

**DOI:** 10.3390/antiox14010074

**Published:** 2025-01-10

**Authors:** Muhammad Junaid Rao, Bingsong Zheng

**Affiliations:** State Key Laboratory of Subtropical Silviculture, College of Forestry and Biotechnology, Zhejiang A&F University, Hangzhou 311300, China

**Keywords:** phenylpropanoid pathway, abiotic stress tolerance, MYB regulatory factors, polyphenols, flavonoids, bioactive molecules, reactive oxygen species, free radicals

## Abstract

Plants have evolved complex mechanisms to cope with diverse abiotic stresses, with the phenylpropanoid pathway playing a central role in stress adaptation. This pathway produces an array of secondary metabolites, particularly polyphenols, which serve multiple functions in plant growth, development, regulating cellular processes, and stress responses. Recent advances in understanding the molecular mechanisms underlying phenylpropanoid metabolism have revealed complex regulatory networks involving MYB transcription factors as master regulators and their interactions with stress signaling pathways. This review summarizes our current understanding of polyphenol-mediated stress adaptations in plants, emphasizing the regulation and function of key phenylpropanoid pathway compounds. We discussed how various abiotic stresses, including heat and chilling stress, drought, salinity, light stress, UV radiation, nanoparticles stress, chemical stress, and heavy metal toxicity, modulate phenylpropanoid metabolism and trigger the accumulation of specific polyphenolic compounds. The antioxidant properties of these metabolites, including phenolic acids, flavonoids, anthocyanins, lignin, and polyphenols, and their roles in reactive oxygen species scavenging, neutralizing free radicals, membrane stabilization, and osmotic adjustment are discussed. Understanding these mechanisms and metabolic responses is crucial for developing stress-resilient crops and improving agricultural productivity under increasingly challenging environmental conditions. This review provides comprehensive insights into integrating phenylpropanoid metabolism with plant stress adaptation mechanisms, highlighting potential targets for enhancing crop stress tolerance through metabolic adjustment.

## 1. Introduction

Challenging ecological and atmospheric conditions pose various environmental challenges to plants during their growth and development, causing significant threats to global agricultural productivity and sustainability [1]. Plants have evolved diverse adaptive mechanisms to ensure their survival under adverse conditions, with the phenylpropanoid pathway and its derived bioactive molecules, particularly polyphenols, playing crucial roles in stress tolerance [2]. This metabolic pathway, extensively studied over the past two decades, produces essential bioactive compounds that facilitate plant adaptation and survival under unfavorable conditions [3]. The phenylpropanoid pathway begins with an amino acid phenylalanine, followed by a complex series of enzymatic reactions, ultimately yielding various classes of bioactive polyphenolic compounds including phenolic acids, flavonoids, anthocyanins, proanthocyanidins, lignin, stilbenes, and hydroxybenzoic acid derivatives [3,4]. These bioactive polyphenol compounds serve multiple functions in plants, from structural support to chemical defense, and, most notably, protection against various abiotic and biotic stresses [5]. Understanding the modulation of the phenylpropanoid pathway under stress conditions has become increasingly important for developing stress-resistant crops, sustainable production, and ensuring food security [3,4,5,6]. This pathway synthesizes more than 8000 different types of bioactive compounds [7,8], which are known for their antioxidant properties and have diverse roles in plant defense. Recent advances in metabolomics and molecular biology have revealed the intricate regulation of this pathway and its responsiveness to unfavorable environmental stimuli [9].

**Figure 1:**
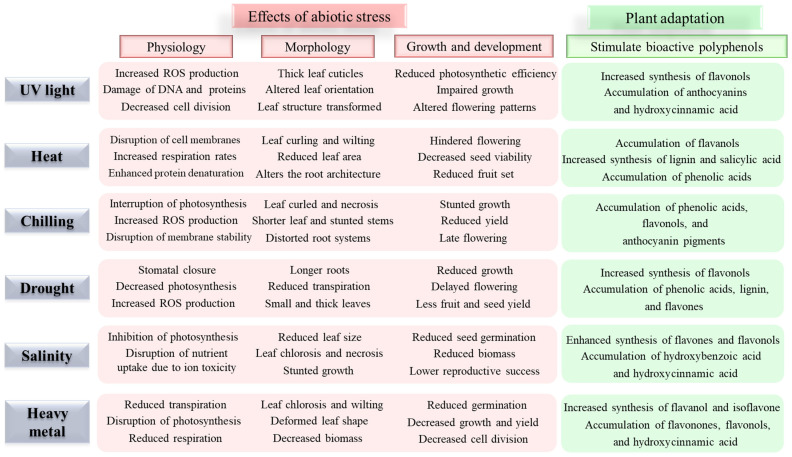
Abiotic stresses affect the physiology, morphology, growth, and development of plants and the plants’ adaptive response by stimulating bioactive polyphenols [3,4,5,6].

Abiotic stresses, including drought, heavy metals, salinity, extreme temperatures, UV light, and oxidative stress, significantly impact plant physiology and agricultural productivity [1,10]. These stresses trigger the production of reactive oxygen species (ROS), leading to oxidative damage of cellular components and impaired physiological functions (Figure 1). To cope with these stresses, plants actively accumulate specific bioactive polyphenolic compounds as part of their stress response mechanism [3,9]. The role of bioactive polyphenols in abiotic stress tolerance is multifaceted [5,6,11,12,13,14,15,16,17]. These compounds function as powerful antioxidants, protecting cellular components from ROS-induced oxidative damage [18,19]. Additionally, polyphenols contribute to membrane stability, osmolyte balance, neutralizing ROS, scavenging free radicals, and the signal transduction pathways involved in stress responses [1,4,5]. Recent studies have demonstrated that different classes of polyphenols exhibit varying levels of effectiveness against specific stresses, suggesting a complex and finely tuned defense mechanism [3,4,6,20].

The regulation of the phenylpropanoid pathway and polyphenol molecule accumulation exhibits significant variation across plant species and among different tissues within the same plant [3]. Environmental factors significantly influence the expression of key enzymes [21,22,23] and the accumulation of specific bioactive polyphenols [1,24,25,26]. This inherent variability presents both challenges and opportunities for research focused on enhancing plant abiotic stress tolerance through the identification and manipulation of potential molecular targets [3,23].

This review aims to integrate current knowledge regarding the modulation of the phenylpropanoid pathway and the role of MYB regulatory factors in regulating polyphenolic compounds to enhance plant stress tolerance under various abiotic stress conditions. We will examine the molecular mechanisms governing pathway regulation, the specific functions of different polyphenol classes in stress responses, specify the individual polyphenolic compounds that accumulate in response to specific abiotic stress, and highlight the role of phenolic compounds in neutralizing the reactive oxygen species and free radicals. This understanding has significant implications for agricultural sustainability. Deciphering these aspects is crucial for developing strategies to enhance crop resilience under increasing environmental challenges.

## 2. The MYB Family Emerges as Particularly Significant in Phenolic Metabolism Regulation and Abiotic Stress Tolerance

MYB transcription factors operate within complex regulatory networks, where individual factors can regulate multiple pathway genes, while single genes may be subject to regulation by MYB proteins [27]. These transcription factors frequently function within larger protein complexes, notably the MBW complex comprising MYB, bHLH, and WD40 transcription factors, and serve as master regulators of flavonoid biosynthesis, specifically in anthocyanin production [27,28,29]. Comprehensive functional characterization of these transcriptional regulators has been achieved within plant species, including *Arabidopsis thaliana*, *Helianthus annuus* L., *Camellia sinensis*, *Medicago truncatula*, *Narcissus tazetta*, and *Vitis vinifera* [30]. The elucidation of regulatory mechanisms governing phenolic metabolism offers substantial potential for enhancing the production of bioactive compounds, thereby improving both abiotic stress tolerance and the yield of medicinally valuable secondary metabolites in plants.

Transcription factors function as specialized proteins interacting with specific promoter regions to stimulate transcription initiation in response to endogenous and exogenous signals, including phytohormones and abiotic stresses [22]. The phenylpropanoid pathway is regulated at the transcriptional and post-transcriptional levels through various molecular mechanisms [22,23]. Multiple transcription factors, including the MYB, bHLH, and WRKY families, serve as key modulators to activate the expression of key enzymatic genes involved in this pathway, thereby accumulating increased levels of bioactive polyphenolic compounds in plant tissues [27]. The MYB family is particularly significant in phenolic metabolism regulation, functioning as an essential mechanism in plant stress responses. Studies have demonstrated that MYB genes exhibit multiple regulatory functions within the phenylpropanoid pathway and flavonoid biosynthesis pathway, serving as both activators and repressors of gene expression [31]. Recent research has revealed the distinct functional roles of MYB transcription factors across various plant species [32]. In the Musa cultivar Rasthal, the overexpression of the *MusaMYB31* gene demonstrates suppressive effects on phenylpropanoid and flavonoid enzymatic pathway genes [33]. Conversely, studies in the *Populus* species identified the *PtMYB115* gene as a positive regulator that promotes proanthocyanidin accumulation through direct interaction with the promoter regions of anthocyanidin reductase 1 (ANR1) and leucoanthocyanidin reductase 3 (LAR3) genes [34]. The *Vitis vinifera VvMYB5a* gene exhibits a unique regulatory mechanism by simultaneously promoting flavonoid synthesis while suppressing lignin production, maintaining metabolic homeostasis between these pathways [32]. The crucial role of MYB transcription factors in regulating secondary metabolite biosynthesis (especially phenolic compounds) and enhancing plant stress tolerance are documented in Table 1. The MYB transcription factors function through multiple regulatory mechanisms, primarily by modulating key enzymes in the phenylpropanoid pathway, including chalcone synthase (CHS), chalcone isomerase (CHI), flavanone-3-hydroxylase (F3H), and flavonol synthase (FLS). These factors orchestrate the accumulation of protective compounds such as flavonoids, anthocyanins, and other phenolic compounds that are powerful antioxidants under abiotic stress conditions (Table 1). Notable examples include AtMYB12, which enhances drought and salt tolerance through flavonoid biosynthesis (Table 1). Stress-protective mechanisms generally involve ROS scavenging, maintaining membrane integrity, and activating stress-responsive genes. This understanding opens new avenues to produce genetically engineered crops with enhanced stress tolerance through the targeted manipulation of MYB transcription factors.

**Figure 2:**
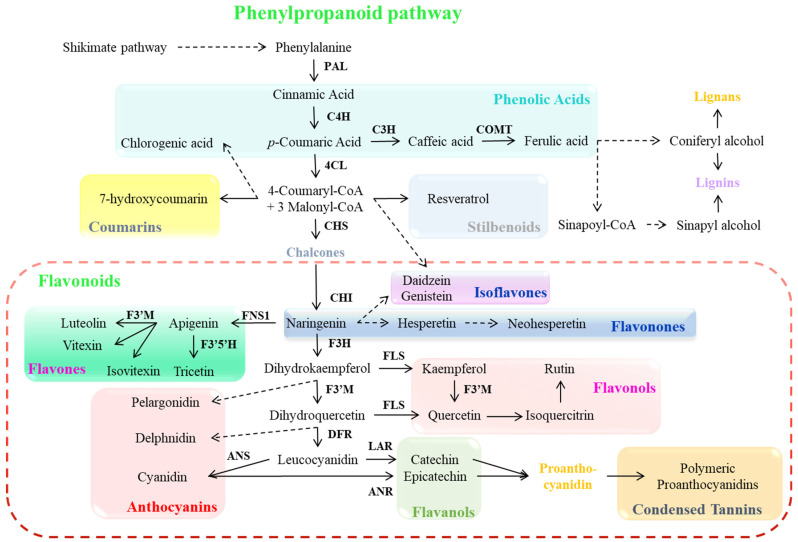
Phenylpropanoid pathway and its biosynthesis genes. Dashed arrows indicate more than one step. Abbreviations: PAL (phenylalanine ammonia lyase), C4H (cinnamate 4-hydroxylase), C3H (cinnamate-3-hydroxylase), COMT (caffeic acid O-methyltransferase), 4CL (4-coumarate: CoA ligase), CHS (chalcone synthase), CHI (chalcone isomerase), F3H (flavanone-3-hydroxylase), FNS1 (flavone synthase 1), FLS (flavonol synthase), F3′M (flavonoid 3′-monooxygenase), F3′5′H (flavonoid 3′5′-hydroxylase), DFR (dihydroflavonol 4-reductase), ANS (anthocyanidin synthase), LAR (leucoanthocyanidin reductase), and ANR (anthocyanidin reductase).

Plant secondary metabolites, such as phenolic compounds, are synthesized through the shikimate pathway, which derives its name from its key intermediate shikimic acid, facilitating the production of aromatic amino acids and phenolic compounds (Figure 2). This pathway initiates with primary metabolites, phosphoenolpyruvate and erythrose-4-phosphate, which combine to form 3-deoxy-D-arabinohexulose-7-phosphate and are then transformed into shikimic acid, ultimately yielding various hydroxybenzoic acids (including p-hydroxybenzoic, protocatechuic, and gallic acids) [3]. This metabolic sequence ends in the biosynthesis of phenylalanine, a crucial amino acid from which the phenylpropanoid pathway begins. The phenylalanine is converted into cinnamic acid by the action of the phenylalanine ammonia-lyase (PAL) enzyme (Figure 2) [61]. The phenylpropanoid pathway proceeds further via the cinnamate 4-hydroxylase (C4H) enzyme reaction that converts cinnamic acid into p-coumaric acid (also known as trans-p-hydroxycinnamate), generating hydroxycinnamic acids, a subgroup of phenolic acids [3,62]. These intermediates undergo various modifications within plant tissues, including β-oxidation to form hydroxybenzoic acids, reduction to produce lignin-associated cinnamic alcohols, formation of acyl derivatives and complex esters like chlorogenic acid, and coumarin synthesis [61,62]. The p-coumaroyl-CoA combines with three malonyl-CoA molecules to form chalcone, catalyzed by chalcone synthase (CHS). Chalcone isomerase (CHI) then converts chalcone naringenin to flavanone naringenin. Following redox modifications of the central heterocyclic ring, naringenin serves as a precursor for diverse flavonoid classes, including flavanols, flavanones, flavones, flavonols, and anthocyanins, though not chalcones or dihydrochalcones [61,62,63]. After that, a series of enzymatic reactions (flavanone-3-hydroxylase, flavone synthase 1, flavonol synthase, flavonoid 3′-monooxygenase, flavonoid 3′5′-hydroxylase, dihydroflavonol 4-reductase, anthocyanidin synthase, leucoanthocyanidin reductase, and anthocyanidin reductase) lead to the production of various phenolic compounds and pigmented anthocyanins and proanthocyanidins (Figure 2) [23,32,62,63].

The biosynthesis of phenolic compounds represents a complex metabolic network where monomeric forms serve as intermediates rather than terminal products. These compounds transform into more complicated oligomeric and polymeric structures [64]. A notable example is the formation of proanthocyanidins, which are complex derivatives synthesized from flavan-3-ol precursors and are widely distributed across plant species [65]. The final phase of the flavonoid pathway involves proanthocyanidins biosynthesis, though the precise mechanisms governing flavanol (such as catechin and epicatechin) condensation into proanthocyanidins remain to be fully elucidated [31]. Current evidence suggests two potential routes for this condensation process: an enzyme-mediated pathway utilizing peroxidase, polyphenol oxidase, and laccase, or a spontaneous non-enzymatic auto-condensation of flavanol units [66]. Recent advances in molecular biology and functional genomics have shifted the research focus toward understanding the genetic basis and regulation of phenolic metabolism, while acknowledging the continued importance of traditional biochemical approaches [31,62]. This molecular perspective has enabled significant progress in genetic engineering strategies aimed at optimizing phenolic profiles in plants, particularly for enhancing the production of bioactive compounds to enhance the abiotic stress tolerance of crops [23].

## 3. The Role of Polyphenolic Compounds in Abiotic Stress Tolerance

Abiotic stresses trigger the production of ROS within plant cells, which demonstrate significant reactivity with different cellular components, such as lipids, nucleic acids, proteins, and cell membranes, potentially leading to cellular dysfunction and death [67]. In response, plants stimulate the biosynthesis of antioxidant secondary metabolites, particularly polyphenolic compounds, as a defense mechanism and adaptive strategy in response to abiotic stresses [68]. These compounds significantly enhance plant tolerance against multiple abiotic stressors, including salinity, heavy metal toxicity, drought, heat stress, chilling injury, UV radiation, and other abiotic stresses [69,70]. The increased synthesis and rapid accumulation of these compounds having potent antioxidative properties during stress conditions can effectively quench the ROS, thereby reducing cellular membrane damage, and serving as a key indicator of plant tolerance and resistance capacity against oxidative stress [71].

Research demonstrates that plants under stress conditions trigger the biosynthesis of polyphenolic compounds compared to those growing in normal conditions [72,73,74,75,76]. The regulation of phenolic compound biosynthesis under stress involves complex enzymatic pathways, with key enzymes such as PAL and CHS playing crucial roles in modulating phenolic synthesis. Under different abiotic stresses, plants regulate multiple genes encoding essential enzymes, including PAL, C4H, C3H, 4CL, COMT, CHS, CHI, F3H, DFR, F3′M, FLS, ANR, and ANS [20]. The elevated expression of these genes results in the enhanced biosynthesis of diverse bioactive polyphenolic compounds, consequently enabling plant resilience through sophisticated stress tolerance mechanisms under adverse environmental conditions.

### 3.1. Temperature

**Figure 3:**
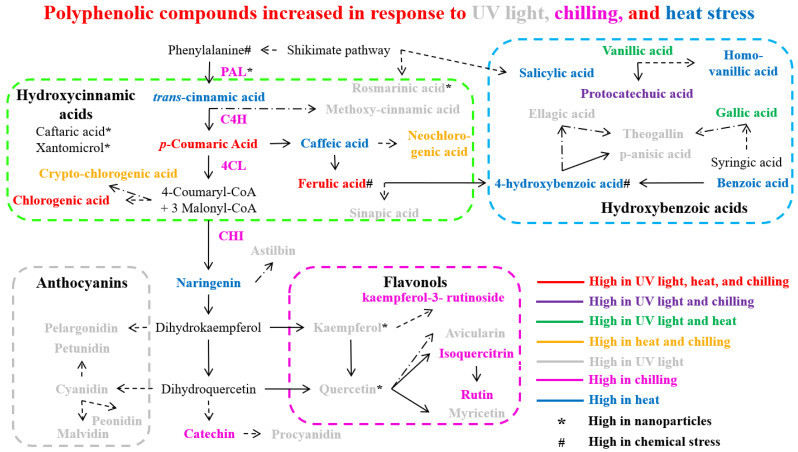
Polyphenolic compounds accumulate in plants in response to abiotic stress such as light stress, temperature stress, nanoparticles, and chemical stress. Dashed arrows indicate more than one step, and dash-dot arrows denote an undefined pathway. Abbreviation: PAL (phenylalanine ammonia lyase), C4H (cinnamate 4-hydroxylase), 4CL (4-coumarate: CoA ligase), and CHI (chalcone isomerase).

Plants exhibit significant adaptability across a vast range of temperature conditions; nevertheless, temperature fluctuations can significantly impact plant morphology, growth, and development throughout the lifecycle [77]. Temperature stress, including heat and chilling conditions, induces plants to synthesize endogenous bioactive phenolic compounds. These compounds include various classes such as phenolic acids, anthocyanins, flavonoids, flavones, and flavonols, which serve as crucial protective molecules in plant cells during stress responses [78,79]. Phenolic compounds are crucial protective biomolecules against temperature-induced stress conditions [80]. Research has shown that chilling or cold stress exposure induces enhanced anthocyanin accumulation in plant tissues [81], concurrent with the increased expression of several flavonoid biosynthesis pathway genes including *PAL*, *C4H*, *4CL*, *CHI*, *DFR*, and *ANS* (Figure 3). *Brassica rapa* has revealed strong associations between cold tolerance and the expression of anthocyanin biosynthesis pathway genes, specifically dihydroflavonol-4-reductase (DFR) and anthocyanidin synthase (ANS) [82,83]. In *Malus sieversii*, low-temperature exposure promotes anthocyanin accumulation through the regulatory action of the MdMYBPA1 transcription factor [65].

Under chilling conditions, increased lignin deposition in epidermal cell layers enhances cell wall rigidity, providing a defense against chilling-induced cellular damage and dehydration [84]. Lignin biosynthesis during extreme temperature stress involves regulatory control by C2H2Zn and MYB transcription factors [85]. During chilling stress exposure, plants increase the production of cell wall-associated phenolics like suberin and lignin, which strengthen cellular barriers against chilling injury [86]. The enhanced cell wall thickness resulting from phenolic deposition helps prevent cellular collapse/damage under chilling conditions [19]. Cold-induced phenolic biosynthesis involves the upregulation of key enzymes including PAL, hydroxycinnamoyl transferase (HCT), and cinnamyl-alcohol dehydrogenase (CAD), and increased level of phenolic compounds, protecting the plant cells from chilling injury [87]. Research on peach trees has shown that 24-epibrassinolide-mediated phenolic accumulation aids in scavenging reactive oxygen species generated during cold stress [78]. In purple head Chinese cabbage (*Brassica rapa* L.) varieties, cold stress activates regulatory genes *BrTT8* and *BrMYB2*, which stimulate the expression of different anthocyanin biosynthesis genes including *BrDFR1*, *BrANS1*, *Br5MAT*, *BrUGT79B1*, and *DrUGT75C1* to accumulate large quantities of anthocyanin pigments in cabbage tissues [88]. In addition, lignin, a dominant complex phenolic polymer in plants, accumulates within cell walls and contributes to chilling tolerance [84].

High temperature or heat stress during plant growth influences antioxidant systems and polyphenol accumulation. In *Solanum lycopersicon*, flavonols accumulated under heat stress can efficiently scavenge the ROS and enhance the *Solanum lycopersicon* adaptability against high-temperature stress [89]. Similarly, elevated temperatures trigger increased flavonoid and phenylpropanoid production in *Glycine max* [90]. Likewise, *Vitis vinifera* revealed temperature-dependent responses varying between day and night cycles, with flavonol content remaining unchanged at 35 °C in darkness and declining at 45 °C regardless of photoperiod [91]. *Festuca trachyphylla* exposed to heat stress revealed significant increases in multiple polyphenols, including vanillic acid, 4-hydroxybenzoic acid, cinnamic acid, caffeic acid, coumaric acid, homovanillic acid, ferulic acid, gallic acid, salicylic acid, and benzoic acid [79]. This enhanced accumulation of phenolics correlates with improved heat stress tolerance [79].

Recent molecular studies of *Chrysanthemum* × *morifolium* cultivar ‘Fencui’ identified CmMYB012 (R2R3-MYB transcription factor), which suppresses flavonoid biosynthesis under prolonged heat exposure by downregulating the expression of flavonoid biosynthesis genes such as *CmCHS*, *CmDFR*, *CmANS*, and *CmUFGT* (UDP-glucose: flavonoid 3-O-glucosyltransferase) [92]. These results showed how different polyphenols respond to temperature stress: chilling conditions generally promote anthocyanin biosynthesis [88], while heat stress typically inhibits these pathways [92,93]. These temperature-mediated changes in polyphenol biosynthesis are governed by the coordinated expression of specific biosynthetic genes and regulatory elements. Research on carrots showed that specific phenolics—coumaric acid, caffeic acid, and anthocyanins—accumulate to reduce heat-induced oxidative damage [79]. Additionally, salicylic acid is a signaling molecule that promotes phenolic biosynthesis during high-temperature stress, thereby enhancing heat resistance by detoxifying ROS [94]. These results showed that plant species modify their phenolic metabolism in response to heat or chilling stress to tolerate unfavorable temperature conditions.

### 3.2. Light

Light represents a fundamental environmental factor that significantly influences plant growth and development [95,96,97]. This crucial factor coordinates with different metabolic pathways, particularly the synthesis of phenolic compounds [75,98,99]. Light regulates several enzymatic genes within the phenylpropanoid pathway, resulting in enhanced production and accumulation of antioxidative polyphenols [63]. The formation of chloroplasts, essential biosynthetic sites for secondary metabolites, is also light-dependent [100]. However, extreme light exposure can simultaneously function as abiotic stress, trigger cellular ROS levels, and potentially initiate oxidative stress responses [101,102].

Multiple studies have revealed a positive correlation between light exposure and antioxidant phenolic compound synthesis within plant tissues. Research has shown that light stimulates the production of various bioactive molecules, including flavonoids, anthocyanins, and proanthocyanidins [75,98,99], in both foliar tissues and *Camellia sinensis* in vitro cultures [103,104]. Notably, a strong correlation was revealed between proanthocyanidin accumulation in leaf tissues and the expression of key phenylpropanoid pathway genes, specifically *PAL*, *F3H*, *F3′H*, *DFR*, and *ANR* [64,66]. Moreover, a high accumulation of O-glycosylated flavonols is associated with the expression of *CHS* and *F3′5′H*, highlighting the complex regulatory network governing polyphenols in response to light [105,106]. The overexpression of the *Citrus sinensis UGT* gene enhanced the total content of flavonoids, anthocyanins, and proanthocyanidins in transgenic *Arabidopsis thaliana* leaves and induced high-light stress tolerance by elevating the antioxidant activity and capacities of transgenic plants [75].

Research has revealed significant interest in understanding how different light wavelengths influence polyphenol biosynthesis and accumulation in plant tissues [63]. Under blue light conditions, *B. napus* and *B. oleracea* have shown increased polyphenol biosynthesis [107]. In *Camellia japonica* (callus cultures), optimal phenols and flavonoid accumulation were achieved by both red-blue or blue-green light combinations [108]. The molecular mechanisms underlying these responses involve light-sensitive transcription factors. Notably, *Fagopyrum tataricum* FtMYB6 (R2R3-MYB transcription factor) exhibits light-induced promoter activity. This factor enhances flavonol biosynthesis by increasing the regulation of FtF3H and FtFLS1 promoters while downregulating the Ft4CL promoter [109].

Plant responses to light are mediated through specialized photoreceptors that detect specific spectral ranges: far-red (700–775 nm), red (620–700 nm), green (500–580 nm), and blue (445–500 nm) [110]. The efficacy of these photoreceptors depends on light intensity and duration [110]. This suggests potential applications in commercial plant cultivation through controlled artificial lighting systems to optimize bioactive polyphenols biomolecule production. Despite extensive research into light-mediated polyphenol accumulation and biosynthesis regulation, the precise mechanisms remain incompletely understood [96,104]. The current understanding primarily covers species–specific responses, which vary considerably based on environmental factors, physiological conditions, and light exposure parameters. This complexity highlights the continuing importance of researching the biochemical and molecular aspects of light-mediated polyphenol biosynthesis in plant biology and biochemistry research.

#### UV Radiation

To date, climate change is one of the most significant environmental challenges facing our planet. Of particular concern is the increasing exposure to harmful solar rays, specifically ultraviolet (UV) radiation, which is stimulated by various factors including greenhouse gas (CO_2_, CH_4_, and N_2_O) emissions, cloud dynamics, and surface albedo from ice and snow cover [111]. The UV light spectrum involves three distinct bands: UV-A (315–400 nm), UV-B (280–315 nm), and UV-C (200–280 nm) [112,113]. UV-A readily penetrates the ozone layer, and UV-C radiation is completely blocked by our planet’s atmosphere. However, UV-B radiation, comprising 5% of total UV radiation, emerges as a significant stress factor for living organisms. UV-B radiation exposure is intense in mountainous regions and areas suffering from ozone depletion. Exposure to UV-B radiation induces changes in plant systems, affecting their physiological, morphological, biochemical, and genetic properties, with responses proportional to radiation strength and duration [112]. Common manifestations include impaired growth, reduced productivity, increased ROS production, decreased photosynthetic capacity, enhanced mutagenesis, and damaged DNA and protein structures (Figure 1). The harmful impact of UV-B radiation displays both immediate and delayed responses linked to altered biosynthetic pathways [114,115].

Interestingly, plants have evolved superior UV-B tolerance compared to other living organisms, largely due to the biosynthesis of protective, biologically active metabolites. Among these, polyphenol antioxidants are prominent due to their dual protective functions: neutralizing or quenching ROS and absorbing short-wavelength radiation, thereby providing metabolic and physical cellular protection [116,117]. For example, when *Rosa damascena* cell cultures were exposed to UV-B radiation they accumulated fifteen-fold flavonoids compared to the control conditions [118]. Similarly, olive leaves exposed to UV-B showed increased production of specific flavonoids like methoxy-luteolin derivatives and elevated levels of β-hydroxy-verbascoside [119]. Furthermore, in *Mangifera indica*, UV-B exposure triggered phenylpropanoid pathway-related genes, where specific chalcone synthase genes (*MiCHS4*, *MiCHS1*, *MiCHS17*) showed enhanced activity [120]. However, plant responses to UV-B radiation depend on inherent phenolic compound levels, their compositional diversity, and tissue distribution patterns.

Plants accumulate the endogenous antioxidant phenolic compounds that form protective shields beneath the epidermal layer. These compounds exhibit significant efficiency in inhibiting thymine dimerization and protecting crucial cellular enzymes, including NAD/NADP, from photooxidative damage. UV-B radiation induces cellular damage in plants through multiple mechanisms, primarily by disrupting protein structures, triggering DNA mutations, and stimulating the production of ROS [121]. In the past few decades, research has shown that plants respond to UV radiation by enhancing flavonoid biosynthesis, resulting in improved UV absorption capabilities and increased radiation tolerance [122]. Flavonoids serve dual protective functions by absorbing both visible light (through pigmented flavonoids such as anthocyanins) and UV radiation (via anthocyanins and non-pigmented flavonoids), effectively protecting the cellular organelles of plants from radiation stress [123]. This adaptation is particularly obvious in high-altitude plants, which synthesize significantly higher concentrations of bioactive polyphenolic molecules than temperate plants [124]. UV radiation stress regulates multiple flavonoid biosynthesis genes, including CHS, CHI, DFR, FLS, F3H, ANS, and PAL [125]. Furthermore, UV light influences polyphenol biosynthesis via both jasmonate-dependent and independent signaling cascades [126], with abscisic acid (ABA) playing a crucial role in regulating UV-induced polyphenols synthesis [127]. These complex molecular mechanisms collectively contribute to plants’ adaptive responses to UV stress.

Various plant species demonstrate significant alterations in phenolic compound profiles under different environmental conditions. In *Arbutus unedo*, elevated levels of specialized phenolics including theogallin, avicularin, and juglanin were observed [128]. Similarly, Broccoli sprouts exhibited an increased accumulation of gallic and sinapic acids [129]. Studies on *Caryopteris mongolica* revealed an enhanced biosynthesis of flavonoids and anthocyanidins, correlating with increased PAL and CHI enzymatic activities [130]. The total content of phenols was increased in tomatoes (*Solanum lycopersicum*) under UV stress [131]. In *Cuminum cyminum*, upregulation of DAHP and PAL gene expression led to higher total phenolic and anthocyanin contents [124]. Analysis of *Fragaria x ananassa* (strawberries) showed the accumulation of kaempferol, ellagic acid, and glucoside derivatives of cyanidin, pelargonidin, and quercetin, accompanied by the upregulation of key flavonoid pathway genes (*CHS*, *CHI*, *DFR*, *FLS*, *UFGT*) [125]. Correspondingly, *Kalanchoe pinnata* displayed elevated levels of total flavonoid and quercitrin content [132]. Studies in *Lactuca sativa* revealed increased levels of phenolic acids (methoxycinnamic, rosmarinic, p-anisic, vanillic, and chlorogenic acids) alongside enhanced PAL activity and expression [133,134]. In addition, the *Ribes nigrum* accumulated higher levels of flavonols, anthocyanins, and phenolic acids after UV-B stress [135]. Studies on *Triticum aestivum* showed temporal changes in phenolic profiles after UV exposure, with increases in ferulic, p-coumaric, and vanillic acids [136], and enhanced the activities of PAL, C4H, 4CL, and COMT [137]. In *Vigna radiata*, elevated flavonoid and phenol contents correlated with increased PAL and CHI activities [138]. *Vitis vinifera* exhibited an increased accumulation of various phenolic compounds including anthocyanins (cyanidin, petunidin, peonidin, and malvidin), flavonoids (quercetin, myricetin, and kaempferol) [139], and phenolic acids (gallic, protocatechuic, and vanillic acids) [127]. The accumulation patterns of bioactive polyphenol compounds (including phenolic acids, flavonoids, and anthocyanins) through the phenylpropanoid biosynthetic pathway in various plant species under UV light stress conditions are presented in Figure 3.

### 3.3. Nanoparticles and Agrochemical Applications

Environmental factors including nanoparticle exposure and agrochemical applications significantly impact phenolic compound metabolism in plants [87,140]. These stressors enhance the biosynthesis of phenolic compounds and play a crucial role in developing stress tolerance mechanisms. The application of various nanoparticles has also been shown to modulate phenylpropanoid metabolism across different plant species. In *Dracocephalum kotschyi*, silicon dioxide nanoparticles enhanced the accumulation of phenols, flavonoids, rosmarinic acid, and xantomicrol, accompanied by an increased expression of PAL and rosmarinic acid synthase (RAS) genes [141]. The titanium nanoparticle treatment of *Vitis vinifera* increased phenolic compounds, particularly caftaric acid and flavonol derivatives including quercetin and kaempferol [142]. Similarly, copper nanoparticles enhanced the accumulation of both phenolic and flavonoid compounds in *Withania somnifera* [140]. Silver nanoparticles induced higher phenolic content in *Solanum Lycopersicon* [143], while zinc nanoparticles elevated phenolic and anthocyanin levels in *Solanum tuberosum* [144]. Agrochemicals such as insecticide application also induce phenolic biosynthetic pathways to accumulate phenolic compounds that contribute to pesticide stress in different plant species [145]. In *Brassica juncea*, insecticide treatment led to the enhanced accumulation of phenolic and polyphenolic compounds [146]. Furthermore, increased levels of phenols and anthocyanins were observed in the leaves of *Brassica juncea* L., concurrent with the upregulated expression of key biosynthetic genes including PAL and chalcone synthase [145,147]. Similar effects were noted in *Oryza sativa*, where insecticide application elevated specific phenylpropanoid pathway metabolites, notably phenylalanine, p-hydroxybenzoic acid, and ferulic acid [148].

### 3.4. Drought Stress

Water deficit or drought conditions trigger ROS production, including both radical forms such as superoxide radical (O2•−), alkoxyl (RO•), and hydroxyl radicals (•OH), as well as non-radical species including singlet oxygen molecules (^1^O_2_) and hydrogen peroxide (H_2_O_2_) compounds [68]. Prolonged drought stress induces elevated levels of reactive oxygen species, specifically hydrogen peroxide and singlet oxygen [20]. These ROS accumulate as products of oxidative stress under drought conditions [149]. Transcriptomic and metabolomic analyses revealed that plants exhibit elevated accumulation of bioactive phenolic compounds, which serve a crucial function in mitigating the adverse effects of drought stress [150]. Water stress conditions induce the biosynthesis of bioactive flavonols, which is directly correlated with increased drought tolerance [151,152,153]. Furthermore, drought conditions modulate the biosynthetic pathways governing phenolic acids, flavonoids, and anthocyanins, resulting in increased levels of antioxidative biomolecules that serve as robust antioxidants to protect plants from drought stress damage [154,155]. Tomato (*Solanum lycopersicum*) plants have shown increased concentrations of specific polyphenols, particularly kaempferol and quercetin, corresponding with enhanced drought resistance [156].

**Figure 4:**
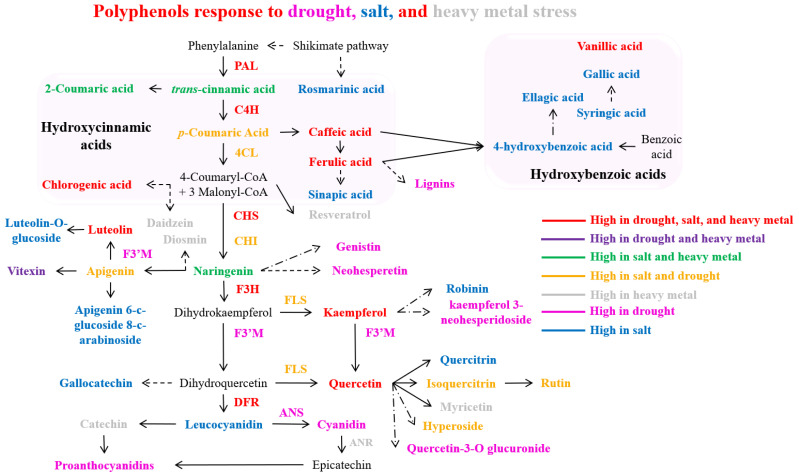
Polyphenol accumulates in response to drought, salt, and heavy metal stress. Dashed arrows indicate more than one step, and dash-dot arrows denote an undefined pathway. Abbreviations: PAL (phenylalanine ammonia lyase), C4H (cinnamate 4-hydroxylase), 4CL (4-coumarate: CoA ligase), CHS (chalcone synthase), CHI (chalcone isomerase), F3H (flavanone3-hydroxylase), FLS (flavonol synthase), F3′M (flavonoid 3′-monooxygenase), DFR (dihydroflavonol 4-reductase), and ANS (anthocyanidin synthase).

The overexpression of the *Citrus sinensis CsCYT75B1* (CYTOCHROME P450) gene increases the total contents of phenolics and flavonoids in transgenic *Arabidopsis thaliana* leaves and induces drought tolerance by elevating the antioxidant properties of transgenic plants [153]. Within plant cells, the increased concentration of flavonoids in the cytoplasm effectively neutralizes drought-induced H_2_O_2_ molecules [156]. These flavonoids undergo oxidation followed by ascorbic acid-dependent reconversion to primary metabolites. This drought-induced polyphenol accumulation primarily results from the activation of several genes that encode essential enzymes associated with the phenylpropanoid biosynthetic pathway, thereby promoting increased phenolic compound synthesis [151,153]. After 21 days of drought exposure, the *Achillea* species exhibited increased concentrations of various phenolic compounds, including luteolin-7-O-glycoside, apigenin, chlorogenic acid, rutin, kaempferol, caffeic acid, luteolin, and 1,3-dicaffeoylquinic acid. This was accompanied by enhanced transcription levels of key enzymes, PAL, CHS, CHI, F3H, F3′H, F3′5′H, and FLS [73], along with elevated total phenols and flavonoid content [157]. *Thymus vulgaris* responded with elevated total flavonoid and polyphenol concentrations [158]. The phenylpropanoid biosynthesis pathway and bioactive polyphenols accumulated in plants under drought stress are illustrated in Figure 4.

During drought stress exposure, the expression of multiple phenylpropanoid pathway biosynthesis genes (*PAL*, *4CL*, *CHS*, *FLS1*, *F3H*, *DFR*, and *ANS*) exhibited a progressive increase in hybrid Populus (*P. tremula × P. alba*) leaves, and, concurrently, a significant increase in the concentration of the salicylic acid (SA) was observed in the drought-stressed poplar leaves [20]. In *Brassica napus*, drought stress induced higher levels of total phenols, flavonoids, and flavonols, concurrent with increased PAL enzyme activity and gene expression [154]. *Chrysanthemum morifolium* demonstrated elevated concentrations of total phenolics, anthocyanins, chlorogenic acid, luteolin, rutin, ferulic acid, apigenin, and quercetin, with enhanced expression of PAL, CHI, and F3H, particularly evident in the Taraneh cultivar [159]. Drought stress in *Cucumis sativus* led to the upregulation of phenolic compounds, notably vanillic acid and 4-hydroxycinnamic acid [155]. *Fragaria ananassa* displayed enhanced transcript levels of PAL, C4H, 4CL, DFR, ANS, FLS, and UFGT [160]. Under drought conditions, *Lactuca sativa* exhibited elevated concentrations of phenolic compounds, particularly caftaric acid and rutin [161]. The Larrea species exhibited elevated levels of polyphenols, including flavonoids, proanthocyanidins, and flavonols [162]. *Lotus japonicus* showed increased kaempferol and quercetin content, accompanied by the upregulated expression of *PAL*, *C4H*, *4CL*, *CHS*, *CHI*, *DFR*, and isoflavone synthase *(IFS*) genes [163]. Under drought stress, *Nicotiana tabacum* demonstrated enhanced PAL enzyme activity and lignin content [164], while the *Ocimum* species showed increased total phenol content [165]. In *Triticum aestivum*, increased total phenol content was observed, along with enhanced concentrations of flavonoids and anthocyanins, accompanied by elevated expression of *CHS*, *CHI*, *F3H*, *FNS*, *FLS*, *DFR*, and *ANS* genes [166]. *Vitis vinifera* (cv. Pinot noir) exhibited increased concentrations of various polyphenols, including 4-coumaric acid, caffeic acid, ferulic acid, cis- and trans-resveratrol-3-O-glucoside, catechin, epicatechin, caftaric acid, epicatechin gallate, kaempferol-3-O-glucoside, cyanidin-3-O-glucoside, quercetin-3-O-glucoside, and quercetin-3-O-glucuronide [17]. Additionally, enhanced anthocyanin content was observed, concurrent with the upregulation of biosynthetic genes including UFGT, CHS, and F3H [167]. To conclude, plant species demonstrated the elevated accumulation of bioactive polyphenols primarily through the activation of genes that encode enzymes associated with the phenylpropanoid pathway in response to water scarcity conditions (Figure 4). This activation resulted in enhanced concentrations of bioactive polyphenols under drought-stress conditions (Figure 4).

### 3.5. Salt Stress

Salt stress in plants occurs when elevated concentrations of sodium (Na^+^) and chloride (Cl^−^) ions are present in soil, which impairs the plant’s water absorption capabilities and causes detrimental effects on plants, including impeded growth, decreased photosynthetic efficiency, disrupted nutrient homeostasis, and, in severe cases, plant mortality (Figure 1) [168]. Salt stress induces the generation of ROS, including radical superoxide, hydrogen peroxide, and hydroxyl ions (OH¯) [169]. This oxidative stress forces plants to activate their antioxidant defense mechanisms to quench ROS [170,171]. This mechanism includes the increased production of bioactive polyphenol molecules that serve as potent antioxidants that effectively scavenge harmful ROS accumulated during salt stress conditions [172,173]. The phenylpropanoid biosynthetic pathway becomes activated under saline conditions, leading to the enhanced production of various phenolic biomolecules with potent antioxidative capabilities [174,175].

Specific genes regulate flavonoid biosynthesis under salt stress conditions [176]. A bHLH transcription factor from *Vitis vinifera*, the *VvbHLH1* gene, enhances the total contents of flavonoids by regulating flavonoid biosynthetic pathway genes, thereby inducing salt tolerance [177]. In tobacco (*Nicotiana tabacum* L.), the *NtCHS1* gene is crucial for flavonoid biosynthesis during salt stress, with increased flavonoid accumulation directly quenching the ROS generated under salt stress. Similarly, salinity stress upregulates the *Glycine max* flavone synthase genes (*GmFNSII-1* and *GmFNSII-2*), promoting flavone biosynthesis and contributing to salinity tolerance [178]. Various phenolic acids, particularly hydroxycinnamic acids and hydroxybenzoic acids, such as vanillic acid, ferulic acid, caffeic acid, gallic acid, cinnamylmalic acid, and caftaric acid, accumulated in response to saline conditions [15,72,179]. Additionally, salt stress promotes the biosynthesis of pigmented flavonoids such as anthocyanin, further enhancing the plant’s antioxidative properties [180,181].

Several plant species improved polyphenol biosynthesis after salinity stress. *Amaranthus tricolor* exhibited increased concentrations of total phenolics, including hydroxybenzoic acids (ellagic, gallic, p-hydroxybenzoic, vanillic, and syringic acids), hydroxycinnamic acids (p-coumaric, caffeic, chlorogenic, m-coumaric, sinapic, ferulic, and trans-cinnamic acids), and flavonoids (hyperoside, iso-quercetin, and rutin) [179]. *Chenopodium quinoa* showed increased bioactive polyphenol and flavonoid content under saline conditions [182]. *Asparagus aethiopicus* demonstrated elevated levels of phenolic compounds, specifically caffeic acid, robinin, apigenin, chlorogenic acid, and rutin, when exposed to salinity stress [175]. *Solanum lycopersicon* showed a significant increment in total caffeoylquinic acid content under salinity stress [171]. The phenylpropanoid pathway and bioactive polyphenols accumulated in plants under salt stress are represented in Figure 4.

*Cynara cardunculus* presented higher concentrations of phenolic compounds, including apigenin 6-c-glucoside 8-c-arabinoside, quercitrin, luteolin-O-glucoside, leucocyanidin, and gallocatechin, while exhibiting decreased levels of ferulic acid, chrysin, daidzein, genistein, and apigenin [183]. Under saline conditions, *Fragaria ananassa* showed the upregulation of several phenylpropanoid pathway genes such as PAL, C4H, F3H, DFR, and FLS [160]. *Salvia mirzayanii* showed an increased total phenolic content [184], while both *S. acrosiphon* and *S. mirzayanii* species revealed enhanced total phenolic content and PAL activity [185]. Under salt stress, *Mentha piperita* and *Hordeum vulgare* exhibited increased total polyphenols contents [186,187]. *Ocimum basilicum* displayed elevated levels of various bioactive polyphenols, including quercetin-rutinoside, caffeic acid, cinnamyl malic acid, rosmarinic acid, feruloyl tartaric acid, and caftaric acid [72] *Olea europaea* showed enhanced concentrations of total phenolics, quercetin, and kaempferol derivatives, accompanied by regulated transcript levels of PAL, C4H, 4CL, CHI, and CHS [174]. *Triticum aestivum* showed increased total phenolic content when exposed to saline stress [188]. *Thymus* spp. exhibited superior concentrations of various phenolic compounds under salt stress, including rosmarinic acid, caffeic acid, quercitrin, trans-2-hydroxycinnamic acid, luteolin, cinnamic acid, gallic acid, rutin, apigenin, naringenin, syringic acid, and vanillic acid [173]. *Solanum villosum* demonstrated elevated total phenolic content, caffeic acid, and quercetin 3-β-D-glucoside levels, along with upregulated expression of PAL and FLS [15]. These findings provide valuable insights into plant adaptation strategies under saline conditions and highlight the potential for enhancing crop stress tolerance through targeted manipulation of phenylpropanoid metabolism.

### 3.6. Heavy Metals Toxicity

Heavy metal toxicity, primarily introduced through anthropogenic activities, represents significant abiotic stressors that adversely affect plant growth and metabolic functions [189]. The severity of heavy metal toxicity is determined by multiple factors, including their chemical properties, bioavailability in soil solutions or growth media, concentration levels, and the duration of metal stress [189,190]. While certain heavy metals such as zinc, copper, and molybdenum are essential micronutrients at low concentrations but become toxic at elevated levels, others like cadmium, lead, and mercury exhibit toxicity even at minimal concentrations due to their non-participation in metabolic processes [190,191,192,193]. Some key genes showed a high expression that encodes phenylpropanoid biosynthesis enzymes including PAL, CHS, F3H, DFR, and ANR. Critical enzymes such as shikimate dehydrogenase (SKDH) and glucose-6-phosphate dehydrogenase (G6PDH) catalyze essential precursor molecules of the phenylpropanoid pathway [194]. The cinnamyl alcohol dehydrogenase (CADH) enzyme enables lignin precursor synthesis, while metal exposure stimulates phenylpropanoid metabolism through enhanced activities of PAL, SKDH, G6PDH, and CADH [195]. Polyphenol oxidase (PPO) contributes to quenching ROS and induces heavy metal stress tolerance [196]. This enzymatic upregulation occurs through transcriptional modulation of corresponding biosynthetic genes under metal stress conditions. Flavonoids contribute significantly to stress tolerance through scavenging H_2_O_2_ and play a significant role in the phenolic/ascorbate-peroxidase cycle [197]. These biochemical adaptations collectively enhance plant resilience under metal stress conditions.

Transcriptomic analysis revealed that *AoMYB12*, *AoWRKY5*, and *AoWRKY6* were involved in Cd heavy metal stress tolerance in *Alisma orientale* [198]. The expression profiling identified several stress-responsive MYB transcription factors in response to cold and cadmium stress in *Apocynum venetum* [199]. Specifically, the *AvMYB48*, *AvMYB97*, *AvMYB8*, and *AvMYB4* genes showed significant transcriptional changes, suggesting their involvement in abiotic stress responses. Additionally, *AvMYB10* and *AvMYB11* were found to regulate proanthocyanidin biosynthesis pathways, displaying functional conservation with their orthologous counterparts in *Arabidopsis thaliana* [199]. Transcriptomic, qPCR, and phylogenetic analyses identified six *BnGMYB* genes (*BnGMYB9*, *BnGMYB10*, *BnGMYB12*, *BnGMYB28*, *BnGMYB41*, and *BnGMYB78*) that exhibited significant responsiveness to cadmium stress conditions [200]. Notably, the *BnGMYB10*, *BnGMYB12*, and *BnGMYB41* genes were significantly upregulated across all plant tissues, with increasing expression levels according to the degree of cadmium stress. Protein interaction network analysis revealed potential molecular interactions between these transcription factors and key regulatory elements involved in flavonoid biosynthesis, suggesting potential mechanistic linkages between cadmium-induced stress responses and the regulation of flavonoid metabolism [200].

Heavy metal stress induces oxidative damage in plants by activating ROS production, causing growth inhibition and cellular toxicity [201]. In response to heavy metal stress, plants enhanced the synthesis of phenolic compounds (served as crucial antioxidants) to protect themselves from metal stress-induced oxidative damage [202,203]. Notably, flavonoids not only neutralize ROS, but, due to metal chelating properties, they form chelate complexes with heavy metals, playing a key role in metal stress tolerance, and effectively reducing metal-catalyzed oxidation reactions within plant cells [204,205]. This chelating ability stems from the nucleophilic nature of their benzene rings, with efficiency varying based on hydroxyl group configuration [193,206]. Notably, polyphenols including flavonoids reveal significant metal-chelating capabilities, as evidenced in *Gynura pseudochina*, where they effectively chelate zinc and cadmium, while catechins specifically chelate iron [207]. In response to cadmium (Cd) stress, plants exhibited increased flavonoid and anthocyanin biosynthesis, accompanied by elevated PAL and CHS expression [208,209]. Prolonged Cd exposure enhanced the accumulation of phenols, polyphenols, and related compounds [209]. The phenylpropanoid pathway and individual bioactive compounds elevated in plants under heavy metal stress are represented in Figure 4.

Studies on *Brassica juncea* have revealed significant modifications in secondary metabolite profiles in response to various metal stresses. Under copper (Cu) exposure, substantial increases were observed in total phenolic compounds, specifically catechin, caffeic acid, coumaric acid, kaempferol, and anthocyanins [197]. Lead (Pb) stress similarly elicited comprehensive increases in phenolic compounds, flavonoids, and anthocyanins, accompanied by corresponding upregulation of PAL and CHS genes [202]. These findings demonstrate consistent patterns of secondary metabolite modulation as a stress response mechanism in B. juncea exposed to heavy metals [208,210]. Red cabbage (*Brassica oleracea*) sprouts exposed to copper metal stress showed concurrent increases in total phenolic content and PAL activity [211]. In wheat (*Triticum aestivum*), both PAL and tyrosine ammonia-lyase activities were enhanced under lead and copper metal stress, with more obvious effects observed with copper stress [212]. Chromium (Cr) stress induced elevated concentrations of phenols, flavonoids, and anthocyanins, coinciding with the enhanced expression of key biosynthetic genes, PAL and CHS. Further investigations of Cr stress confirmed anthocyanin accumulation, which specifically correlated with CHS gene upregulation [213]. The regulatory transcription factors, particularly the MYB family, with the R2R3MYB subfamily, play a crucial role in stress responses through the MBW complex-mediated regulation of flavonoid and anthocyanin biosynthesis [189,190,214]. In *Artemisia annua*, the overexpression of metabolite enzymatic genes enhances artemisinin production under copper and silver stress, while elevated PAL and CHS gene activity increases flavonoid and anthocyanin levels [207].

Multiple plant species exhibit enhanced phenolic metabolism under metal stress conditions. In *Fagopyrum esculentum*, aluminum exposure led to elevated phenolic compounds, flavonoids, anthocyanins, and PAL activity [215]. Similarly, *Kandelia obovata* showed increased phenolic accumulation when exposed to cadmium and zinc, accompanied by the upregulation of key enzymes including shikimate dehydrogenase, cinnamyl alcohol dehydrogenase, and polyphenol oxidase [216]. Copper stress in *Vitis vinifera* induced transcriptional changes in phenylpropanoid pathway genes, upregulating PAL, C4H, CHS, F3H, and DFR while suppressing UFGT and ANR expression [217]. Cadmium exposure in *Withania somnifera* enhanced flavonoid and phenolic accumulation [195]. In *Zea mays*, multiple heavy metals (Cu, Pb, and Cd) triggered increases in total phenols and specific compounds like chlorogenic and vanillic acids [214]. Heavy metal stress increased catechins and quercetin levels in *Pinus* and *Zea mays* root systems [218]. Similarly, copper stress triggered flavonoid production in *Amaranthus caudatus* and *Ginkgo biloba* callus cultures [219]. Under lead stress, *Prosopis farcta* displayed higher phenolic content and PAL activity, with increases in specific compounds like ferulic acid, cinnamic acid, caffeic acid, and various flavonoids including daidzein, vitexin, resveratrol, and quercetin [220]. However, some phenolic compounds such as anthocyanin significantly decreased after excessive nickel stress in *Lactuca sativa* sprouts, while certain Asteraceae family members showed reduced secondary metabolite production under metal stress [19]. This reduction likely results from severe heavy metal toxicity including both antioxidant systems and metabolite biosynthesis pathways. These studies revealed that the activation of phenylpropanoid pathways correlates with increased heavy metal stress tolerance in plants.

### 3.7. Polyphenols Pathway Responds to Multiple Environmental Stressors

The complex interrelationships between individual abiotic stresses and their regulatory effects on polyphenol metabolism across diverse plant species have been extensively documented (Figure 3 and Figure 4). Notably, under natural or field conditions, plants rarely experience isolated stress factors. Rather, they are typically subjected to multiple concurrent environmental stresses, resulting in distinct metabolic responses that differ significantly from those observed under single-stress conditions. In *Eucalyptus globulus*, combined drought and heat stress led to significantly elevated levels of cinnamate (trans-cinnamic acid), whereas individual drought or heat stress did not produce comparable increases [221]. Given that cinnamate serves as a precursor for various polyphenols, elevated concentrations of this compound may generate diverse phenylpropanoids that potentially contribute to stress tolerance against combined drought and heat conditions in *E. globulus* [221]. Interestingly, under combined drought and heat stress, total anthocyanin content remained relatively stable in both *Pinus elliottii* var. elliottii and the hybrid *Pinus elliottii* var. elliottii × *Pinus caribaea* var. hondurensis [222]. These findings underscore the importance of investigating stress combinations rather than individual stress; such studies would significantly enhance our understanding of plant adaptation mechanisms in natural environments.

Under combined drought and heat stress conditions, the ‘Cobrançosa’ olive (*Olea europaea*) cultivar exhibited reduced levels of luteolin-4-O-glucoside, apigenin-7-O-glucoside, and quercetin-3-rutinoside compared to control conditions [223]. However, p-coumaric acid levels increased by more than 70% under combined stress conditions. While drought and heat stress individually enhance the accumulation of phenolic compounds in plants (Figure 3 and Figure 4), the polyphenols decreased under combined stress conditions in olives, except p-coumaric acid [223]. These findings suggest that the combination of drought and heat stress may operate through distinct mechanisms, potentially by suppressing endogenous enzymes such as oxidoreductases, polyphenol oxidase, and peroxidase, which are responsible for phenolic compound oxidation [224], or by downregulating polyphenol-related genes including PAL, CHS, and dihydroflavonol reductase [225]. Notably, under combined stress conditions, the ‘C.C.Branco’ olive cultivar demonstrated suppressed flavonoid production while accumulating elevated levels of oleuropein, another bioactive compound [223]. These metabolic modifications underscore the complex nature of stress responses across different olive cultivars and suggest the existence of diverse adaptive mechanisms within the species. This phenomenon highlights the importance of investigating stress combinations rather than isolated stresses to gain a more comprehensive understanding of plant adaptation mechanisms in natural environments.

## 4. Polyphenols Antioxidant Properties to Scavenge Free Radicals and Reactive Oxygen Species

Under abiotic stress conditions, various phenolic compounds play crucial roles in enhancing plant resilience by exhibiting antioxidant activity during abiotic stress or unfavorable environmental conditions [19]. The effectiveness of polyphenols to scavenge free radicals or reactive oxygen species can vary based on the type of abiotic stress (e.g., drought, salinity, or heat), duration, and specific plant species [5,67]. In our review, we have found that several phenolic compounds such as quercetin, kaempferol, luteolin, apigenin, naringenin, ferulic acid, chlorogenic acid, and caffeic acid commonly respond to different abiotic stresses in various plant species (Figure 3 and Figure 4). Among these, quercetin is one of the most potent antioxidants in plant species and can effectively quench the ROS and mitigate oxidative stress caused by abiotic factors. Quercetin donates hydrogen atoms to free radicals, especially superoxide anions, hydroxyl radicals, and hydrogen peroxide, and inhibits lipid peroxidation [226]. Thus, stabilizing the free radicals enhances plant tolerance to various stresses and prevents plants’ cellular organelles from oxidative damage [226]. Like quercetin, kaempferol also exhibits strong antioxidant properties, enhances the activity of endogenous antioxidant enzymes, reduces ROS levels, and protects plants from oxidative damage, thereby improving abiotic stress tolerance [227,228,229,230]. Kaempferol acts as a hydrogen donor, and high biosynthesis of this compound under abiotic stress can efficiently neutralize the superoxide and hydroxyl radicals during abiotic stress [228,231,232].

Luteolin and apigenin are recognized for their antioxidant properties that scavenge free radicals (superoxide radicals and hydroxyl radicals by donating hydrogen), which have been shown to improve plant resilience under abiotic stress conditions [233,234]. Additionally, luteolin also chelates metal ions, which may contribute to its antioxidant activity by preventing the Fenton reaction that generates hydroxyl radicals; besides this, it has a wide range of bioactivities, including anti-inflammatory, antioxidant, antibacterial, neuroprotective, antiviral, and cardioprotective effects [204,233]. Naringenin exhibits potent antioxidant properties through its ability to donate hydrogen atoms, neutralize superoxide anions, and scavenge hydroxyl radicals and hydrogen peroxide molecules [232,235]. Ferulic acid levels increased in several plant species in response to different abiotic stresses such as drought, heat, chilling, salt, UV light, and heavy metal stress (Figure 3 and Figure 4). Ferulic acid exhibits antioxidant activity by hydrogen donation to scavenge the hydroxyl radicals and peroxyl radicals (ROO•) and triggers the activity of other antioxidants [236,237]. Chlorogenic acid is a robust antioxidant that can scavenge free radicals by donating electrons and hydrogen atoms to neutralize free radicals (hydroxyl radicals, hydrogen peroxide, and superoxide radicals) produced during abiotic stress [238]. Caffeic acid has moderate antioxidant activity compared to the phenols mentioned above. It can stabilize the superoxide radicals and make them least reactive to protect them from oxidative damage during plant abiotic stress [239]. Among these compounds, quercetin, kaempferol, and luteolin are often recognized for their high antioxidant activity, followed by naringenin, chlorogenic acid, and ferulic acid, which also showed significant antioxidant properties, protecting plants from oxidative damage, and contribute to stress tolerance; however, apigenin and caffeic acid have moderate antioxidant activities [204,232,237,239]. However, the efficacy of these phenols can depend on the plant species and type of abiotic stress.

## 5. Conclusions

This review highlights the crucial role of polyphenolic compounds in plant stress tolerance mechanisms. The phenylpropanoid pathway emerges as a central metabolic route that produces diverse bioactive polyphenols essential for plant adaptation to various environmental stresses. These compounds exhibit significant antioxidant properties, effectively neutralizing reactive oxygen species and free radicals generated during abiotic stress. MYB transcription factors have been identified as master regulators of phenylpropanoid metabolism, controlling the expression of key biosynthetic genes and subsequently influencing polyphenol accumulation patterns under stress conditions. The regulatory networks involving these transcription factors represent promising targets for enhancing crop stress tolerance through genetic engineering approaches. This review reveals that plants accumulate specific phenolic compounds in response to different abiotic stresses, with some compounds like quercetin, kaempferol, and luteolin showing consistently high antioxidant activity across various stress conditions. However, plant responses to combined stresses often differ from single-stress scenarios, highlighting the complexity of stress adaptation mechanisms in natural environments. Understanding these intricate regulatory mechanisms and stress response patterns provides valuable insights for developing stress-resistant crops. Future research should focus on elucidating the precise molecular mechanisms governing polyphenol-mediated stress tolerance and exploring the potential of targeted genetic modifications to enhance crop resilience in the face of increasing environmental challenges.

## Figures and Tables

**Table 1:** Role of MYB transcription factors as master regulators of secondary metabolism and abiotic stress tolerance in plants.

Serial No.	Transcription Factor	Plant Species	Regulated Compounds	Stress Tolerance	Regulatory Mechanism	Stress-Protective Mechanism	References
1	*AtMYB12*	*Arabidopsis thaliana*	Flavonoids and flavonols	Salt and drought	Activation of CHS, CHI, and F3H genes	Neutralization of ROS	[35]
2	*AtMYB12*	*Arabidopsis thaliana*	Flavonoid and flavones	UV-B	Upregulates FLS and CHS genes	Protect photosynthetic apparatus	[36]
3	*GbMYB11*	*Ginkgo biloba*	Flavonol	Salt	F3′H and FLS	Neutralize ROS	[37]
4	*MdMYB2*	Apple Borkh	Anthocyanins	Cold tolerance	Anthocyanin biosynthesis genes	Interact MdSIZ1 promoter	[38]
5	*MdMYB88*	*Malus domestica*	Anthocyanidins, kaemferol, and quercetin	Salt and cold stress	Regulates MdUGT83L3	Decreased ROS	[39]
6	*MdMYB308L*	*Malus domestica*	Accumulates anthocyanins	Cold	Binds to promoters of the DFR gene	Interaction with MdbHLH33	[40]
7	*VyMYB24*	*Vitis yanshanesis*	Regulates proline, gibberellin, and metabolites	Drought tolerance	Enhances SOD, POD, and CAT	Decreased ROS	[41]
8	*VvMYBF1*	*Vitis vinifera* L.	Flavonoid	Drought and salt	Regulates PAL, CHI, FLS, and DFR.	Reduction of H_2_O_2_ and MDA	[42]
9	*VhMYB15*	hybrid *V. riparia* L. and *V. labrusca*	Proline and antioxidant enzymes	Salinity and drought	Regulates AtSOS1-3 and AtNHX1	Less H_2_O_2_ and MDA, high SOD, POD, CAT	[43]
10	*MYB4*	*Arabidopsis thaliana*	Negative regulator of sinapate ester and flavonoids	UV-B stress	Represses cinnamate 4-hydroxylase (C4H)	Repair UV-B-induced DNA breaks	[44]
11	*AtMYB111*	*Arabidopsis thaliana*	Increases quercetin production	Salt stress response	Regulates CHS, F3H, and FLS1 genes	Salt resistance	[45]
12	*GbMYB1*	*Ginkgo biloba*	Activates flavonoid biosynthesis	UV-B radiation	Regulates FLS module	Photoprotection by anthocyanins	[46]
13	*VaMyb14*	*Vitis amurensis*	Activating lipid transfer protein	Cold and drought tolerance	Controls stilbene synthase expression	Increased POD activity and reduced MDA	[47]
14	*VviMYB24*	Cabernet Sauvignon’ grape	Positively regulates flavonol biosynthesis	Drought stress	Activate VviFLS5 expression	Forms MYB24-iMYC2b-FLS5 module	[48]
15	*MYB-Arahy.J3K16K*	*Arachis hypogaea*	Increases anthocyanins	Abiotic stress tolerance	Regulates PAL, CHS, CHI, F3H, DFR, and ANR	MAPK cascade, ethylene, auxin, and abscisic acid	[49]
16	*LlMYB3*	*Lilium lancifolium* L.	Anthocyanin accumulation	Chilling, drought, and salt	Directly binds with LlCHS2	Upregulates APX2 and LEA14	[50]
17	*LhMYBC2*	Lilium cv. ‘Sunny Martinique’	Modulates flavonoid biosynthesis	Heat stress	Regulates isoflavonoid biosynthesis genes	Increased isoflavonoid	[51]
18	*CsMYBL2*	*Camellia sinensis*	Upregulates anthocyanin and catechins	Light and temperature	Activates phenylpropanoid biosynthesis genes	Enhances cold and UV-B tolerance	[52]
19	*RsMYB1*	*Raphanus sativus*	Enhances anthocyanin production	Heavy metal stress	Activates anthocyanin biosynthesis genes	Increased activity of GST, PCS, SOD, POD, and CAT	[53]
20	*SlMYB14*	*Solanum lycopersicum*	Promotes flavonoid biosynthesis	Pesticide stress	Binds with SlPAL gene	Maintain ROS homeostasis	[54]
21	*SsMYB113*	*Schima superba*	Accumulates flavonoids	Drought	Bind directly to *SsCHS* gene	Modulates ROS generation	[55]
22	*FtMYB11*	*Fagopyrum tataricum*	Flavonoids	Drought, Salt, and ABA	Regulates AtC4H, AtF3H, AtANS, AtFLS, and At4CL	Regulating MDA and proline	[56]
23	*VvmybA1* from *Vitis vinifera*	Overexpressed in Hamlin’s citrus trees	Anthocyanin accumulation	Cold stress	Regulates anthocyanin biosynthesis genes	Enhanced ROS scavenging capacity	[57]
24	*GmMYB12*	*Glycine max*	Flavonoids accumulation	Salt and drought	Upregulates flavonoid genes	Increase proline content, SOD, and POD, less MDA and H_2_O_2_	
25	*ScAN2* (MYB)	*Solanum commersonii*	Activates hydroxycinnamic acid derivatives	Cold stress	Regulates phenylpropanoid genes	Quench ROS and induce cold tolerance	[58]
26	*AtMYB2*	*Arabidopsis thaliana*	Triggers phenolic acids synthesis	Salt stress	Regulates phenolic acid genes	Decrease ROS accumulation	[59]
27	*AtMYB3*	*Arabidopsis thaliana*	Stimulates lignin and anthocyanins	Salt stress	Promote expression of PAL1, C4H, COMT, DFR, etc.	Reduces ROS	[60]

*Solanum lycopersicum* phenylalanine ammonia-lyase (SlPAL), *Arabidopsis thaliana* cinnamate 4-hydroxylase (AtC4H), anthocyanidin synthase (ANS), 4-coumarate: CoA ligase (4CL), caffeic acid O-methyltransferase (COMT), flavonoid 3′-hydroxylase (F3′H), dihydroflavonol reductase (DFR), *Malus domestica* UDP-glucosyltransferase 83L3 (MdUGT83L3), *Malus domestica* sap and miz1 domain-containing ligase1 (MdSIZ1), glutathione S-transferase (GST), phytochelatin synthase (PCS), bHLH transcription factor (VviMYC2b), flavonol synthase 5 (FLS5), late embryogenesis abundant 14 (LEA14), hydrogen peroxide (H_2_O_2_), malondialdehyde (MDA), *Arabidopsis thaliana* Salt Overly Sensitive 1-3 (AtSOS1-3), *Arabidopsis thaliana* Na+/H+ exchanger 1 (AtNHX1), superoxide dismutase (SOD), catalase (CAT), ascorbate peroxidase 2 (APX2), and peroxidase (POD).

## Data Availability

All the data are available in the main text.
